# Intra-individual comparison between abdominal virtual mono-energetic spectral and conventional images using a novel spectral detector CT

**DOI:** 10.1371/journal.pone.0183759

**Published:** 2017-08-24

**Authors:** Jonas Doerner, Christian Wybranski, Jonathan Byrtus, Christian Houbois, Myriam Hauger, Carola Heneweer, Florian Siedek, Tilman Hickethier, Nils Große Hokamp, David Maintz, Stefan Haneder

**Affiliations:** Institute of Diagnostic and Interventional Radiology, University Hospital of Cologne, Cologne, Germany; Lee Kong Chian School of Medicine, SINGAPORE

## Abstract

**Objectives:**

To quantitatively and qualitatively assess abdominal arterial and venous phase contrast-enhanced spectral detector computed tomography (SDCT) virtual mono-energetic (MonoE) datasets in comparison to conventional CT reconstructions provided by the same system.

**Materials and methods:**

Conventional and MonoE images at 40–120 kilo-electron volt (keV) levels with a 10 keV increment as well as 160 and 200 keV were reconstructed in abdominal SDCT datasets of 55 patients. Attenuation, image noise, and contrast- / signal-to-noise ratios (CNR, SNR) of vessels and solid organs were compared between MonoE and conventional reconstructions. Two readers assessed contrast conditions, detail visualization, overall image quality and subjective image noise with both, fixed and adjustable window settings.

**Results:**

Attenuation, CNR and SNR of vessels and solid organs showed a stepwise increase from high to low keV reconstructions in both contrast phases while image noise stayed stable at low keV MonoE reconstruction levels. Highest levels were found at 40 keV MonoE reconstruction (p<0.001), respectively. Solid abdominal organs showed a stepwise decrease from low to high energy levels in regard to attenuation, CNR and SNR with significantly higher values at 40 and 50 keV, compared to conventional images. The 70 keV MonoE was comparable to conventional poly-energetic reconstruction (p≥0.99). Subjective analysis displayed best image quality for the 70 keV MonoE reconstruction level in both phases at fixed standard window presets and at 40 keV if window settings could be adjusted.

**Conclusion:**

SDCT derived low keV MonoE showed markedly increased CNR and SNR values due to constantly low image noise values over the whole energy spectrum from 40 to 200 keV.

## Introduction

The potential of virtual mono-energetic imaging (MonoE) derived from dual energy computed tomography (DECT) to increase iodine attenuation at low kilo-electron volt (keV) levels has been exploited to enhance signal- and contrast-to-noise ratios (SNR and CNR) in DECT angiography (DE-CTA) studies [1–3]. Its capability to reduce beam hardening and scatter artifacts at high keV levels has been utilized to balance image noise from metal stents [1, 4–9]. Previously published studies have demonstrated the superior diagnostic accuracy of DE-CTA compared to single energy CT angiography (SE-CTA) for the assessment of vessel stenosis [10, 11]. However, because of its inherent technical features MonoE at low energy levels show high noise levels and in consequence impaired image quality, which might be improved by vendor specific algorithms [12].

MonoE derived from DECT are essentially calculated as a linear combination of the high- and low-energy photons of the poly-energetic x-ray spectrum, simulating as if imaged with a true mono-energetic x-ray spectrum [13]. Until recently, commercially available DECT-scanners acquired these high- and low-energy datasets by modulation of the x-ray tube voltage or beam hardening, either by a) two consecutive rotations of a single x-ray tube at different potentials (dual-spin), b) two independent orthogonally positioned x-ray tube-detector systems with different potentials (dual-source), c) splitting of the output of a single x-ray tube by means of a beam filter resulting in two partial beams with different energies (split or twin beam), or d) rapid switching of the potential of a single x-ray tube during a single rotation (kV_p_ switching) [14, 15].

Contrary to the aforementioned emission-based technologies, spectral detector computed tomography (SDCT) is a detector-based solution. It employs a single poly-energetic x-ray source and despite an overlap of the spectral responses, the low-energy x-ray photons of the poly-energetic spectrum are preferably absorbed in the surface layer–an yttrium-based garnet scintillator–and the high-energy photons respectively in the bottom layer–a gadolinium oxysulphide scintillator [14, 16]. Consequently, this technical approach allows the simultaneous measurement of low and high-energy photons at the exact same spatial and angular location facilitating dual-energy post-processing in the projection domain, different to other dual-energy techniques [17, 18]. Furthermore these simultaneous measurements allow to use the noise anti-correlation between the two detector layers providing unique opportunities for noise identification and suppression [19]. This may theoretically lead to lower noise at low energy levels. Overall this should improve the diagnostic imaging quality of very low keV images and lead to a lower and more uniform noise distribution across the energy spectrum.

So far, the evaluation and intra-individual comparison of MonoE derived by SDCT with poly-energetic (conventional) images in arterial and venous phase had not been performed. Therefore, this study aimed to assess quantitative and qualitative image parameters of MonoE in abdominal arterial and venous phase contrast-enhanced SDCT datasets in comparison to conventional CT reconstructions of the same scan.

## Materials and methods

### Study population

Approval from the ethics committee of the University of Cologne, Faculty of Medicine, was obtained for this retrospective study and requirement to obtain written informed consent was waived. The study population comprised 55 patients (33 male, 22 female) with a mean age of 65.6 ± 13.2 years (range 28–83 years) who were referred to oncological follow-up imaging between June and August 2016. The underlying diagnoses of the patients were: malignant melanoma (n = 25), esophageal cancer (n = 12), sarcoma (n = 4), breast cancer (n = 3), renal/transitional cell cancer (n = 2), gastrointestinal stromal tumor (n = 2), lung cancer (n = 2), pancreatic carcinoma (n = 2) neuroendocrine tumor (n = 2) and others (each n = 1).

### Image acquisition and post-processing

All examinations were performed using a SDCT scanner (IQon, Philips Healthcare, Best, The Netherlands). Patients were positioned supine and scanned in cranio-caudal direction during breath-hold. The clinical routine protocol for oncological follow-up comprised arterial phase imaging of the upper and venous phase imaging of the complete abdomen including the pelvis. Images were obtained 20 s and 70 s after a bolus application of 120 ml non-ionic, iodinated contrast media (Accupaque 350 mg/ml, GE Healthcare; Little Chalfort, UK) injected via an antecubital vein at a flow rate of 3.5 ml/s followed by a 30 ml saline chaser. For contrast media timing the bolus-tracking technique was activated in all cases, starting the examination with the aforementioned scan delay after a trigger threshold of 100 Hounsfield units (HU) had been reached within a region of interest (ROI) placed in the abdominal aorta just below the diaphragmatic dome. The following scanning parameters were kept constant in all scans: collimation—2 x 64 x 0.625 mm; rotation time—0.5 s; pitch—0.671; tube current—120 kVp, matrix—512 x 512; dose modulation type: DoseRight 3D-DOM (Philips Healthcare, Best, The Netherlands). All axial images were reconstructed with a slice thickness of 2 mm and a section increment of 1 mm using a dedicated spectral reconstruction algorithm with a strength level of 3 and a constant kernel (Spectral B, Philips Healthcare, Best, The Netherlands). In addition to the conventional 120 kilo-volt (kV) images, 11 MonoE datasets were reconstructed from the arterial and venous phase images using 10 keV intervals from 40–120 keV as well as 160 and 200 keV. Image analysis was performed offline on a dedicated workstation (IntelliSpace Portal 6.5, Philips Healthcare, Best, The Netherlands).

### Quantitative image analysis

Two readers (each 5 years of experience in abdominal CT) in consensus placed circular ROIs in the supra- and infra-renal abdominal aorta, coeliac trunk, common hepatic artery (CHA), splenic artery (SA), superior mesenteric artery (SMA), as well as left and right renal artery (RA) in all datasets acquired in both contrast phases. The ROIs were drawn as large as possible within the center of the vessels, excluding vessel walls as well as areas of stenosis or calcification if present.

In the datasets acquired in venous phase, additional circular ROIs were placed in the portal vein (PV), liver, pancreas, and in the cortex of the left and right kidney. Potentially disturbing structures like metastasis, artifacts, parenchymal vessels, etc. were carefully excluded. The size of the ROIs in the parenchymal organs varied between 100–300 mm^2^. For each ROI, absolute attenuation values in Hounsfield units (HU) as well as the standard deviation (SD) were recorded. All vascular measurements were performed twice and all organ measurements in triplicate. Repetitive measurements and both kidneys were averaged for each patient.

Additionally, in all scans a circular ROI was placed in the psoas muscle and the retroperitoneal fat as a surrogate for image noise and for calculation of the contrast to noise ratio (CNR). CNR of vessels and solid organs were calculated using the following formula:

$$CNR=(HU_{artery/solid\;organ}-HU_{muscle})/image\;noise$$

Image noise was defined as the SD of fat. The signal to noise ratio (SNR) was defined as [6, 8, 20]:

$$SNR=HU_{artery/solid\;organ}/SD_{artery/solid\;organ}.$$

### Qualitative image analysis

The same two radiologists assessed the subjective image quality for all datasets four weeks after the objective reading. To validate the scale of the qualitative image analysis, training data sets were evaluated by the two radiologists in a consensus reading and corresponding scale grading was defined. All datasets were then assessed independently and in a blinded, random order for reconstruction level and contrast phase, respectively. Images were evaluated for contrast conditions, detail visualization, and overall image quality, and compared to the conventional reconstructions using a 5-point Likert-scale, respectively. Grading for contrast conditions was defined as: -2 = substantially lower contrast, -1 = lower contrast, 0 = same contrast, 1 = enhanced contrast, 2 = markedly enhanced contrast. Grading for detail visualization was defined as: -2 = substantially impaired detail visualization, -1 = mild impaired detail visualization, 0 = same detail visualization, 1 = increased detail visualization, 2 = markedly increased detail visualization. Grading for overall image quality was defined as: -2 = severely impaired image quality due to excessive image noise and/or poor conspicuity of vessel walls, -1 = fair–impaired image quality due to substantial image noise and/or poor conspicuity of vessel walls, limitations in low contrast resolution are evident, 0 = same image quality, 1 = increased image quality due to lower image noise and clear conspicuity of vessel walls, 2 = best image quality with only minimal perception of image noise, no limitations in low contrast resolution, excellent attenuation of the vessel lumen and clear conspicuity of the vessel walls.

Fixed common standard window settings for abdominal imaging with a center of 60 HU and a width of 360 HU were used. Reviewers were not allowed to adjust window settings in this session in order to maintain comparable conditions.

A second reading was performed in 25 randomly chosen MonoE datasets at reconstruction levels of 40, 50, and 70 keV, and the corresponding conventional images to assess subjective image noise and overall image quality. In this reading, reviewers were allowed to freely adjust window settings to their personal preferences. Subjective image noise was rated by a 5-point Likert scale with the following criteria ranging from 1 = excessive image noise to 5 = very low image noise with sharp images. Additionally, overall image quality was reevaluated with a 5-point Likert scale ranging from 1 = low image quality, not suitable for diagnostic reading to 5 = excellent image quality.

### Statistical analysis

Statistical analysis was performed using GraphPad Prism (version 7.0b for Macintosh, GraphPad Software, La Jolla California USA). Descriptive statistics are summarized as means ± SD. For continuous variables, one-way ANOVA followed by Tukey’s multiple comparisons post-hoc test was performed. Statistical significance was defined as p ≤ 0.05. Wilcoxon signed-rank test was performed to compare the qualitative image parameters. Inter-reader agreement was calculated with quadratic weighted Cohen's kappa coefficients (κ), with values of ≥ 0.81 indicating excellent, 0.61–0.80 substantial, 0.41–0.60 moderate, 0.21–0.40 fair, and ≤0.20 poor agreement.

## Results

### Quantitative image analysis

#### Attenuation

**Fig 1:**
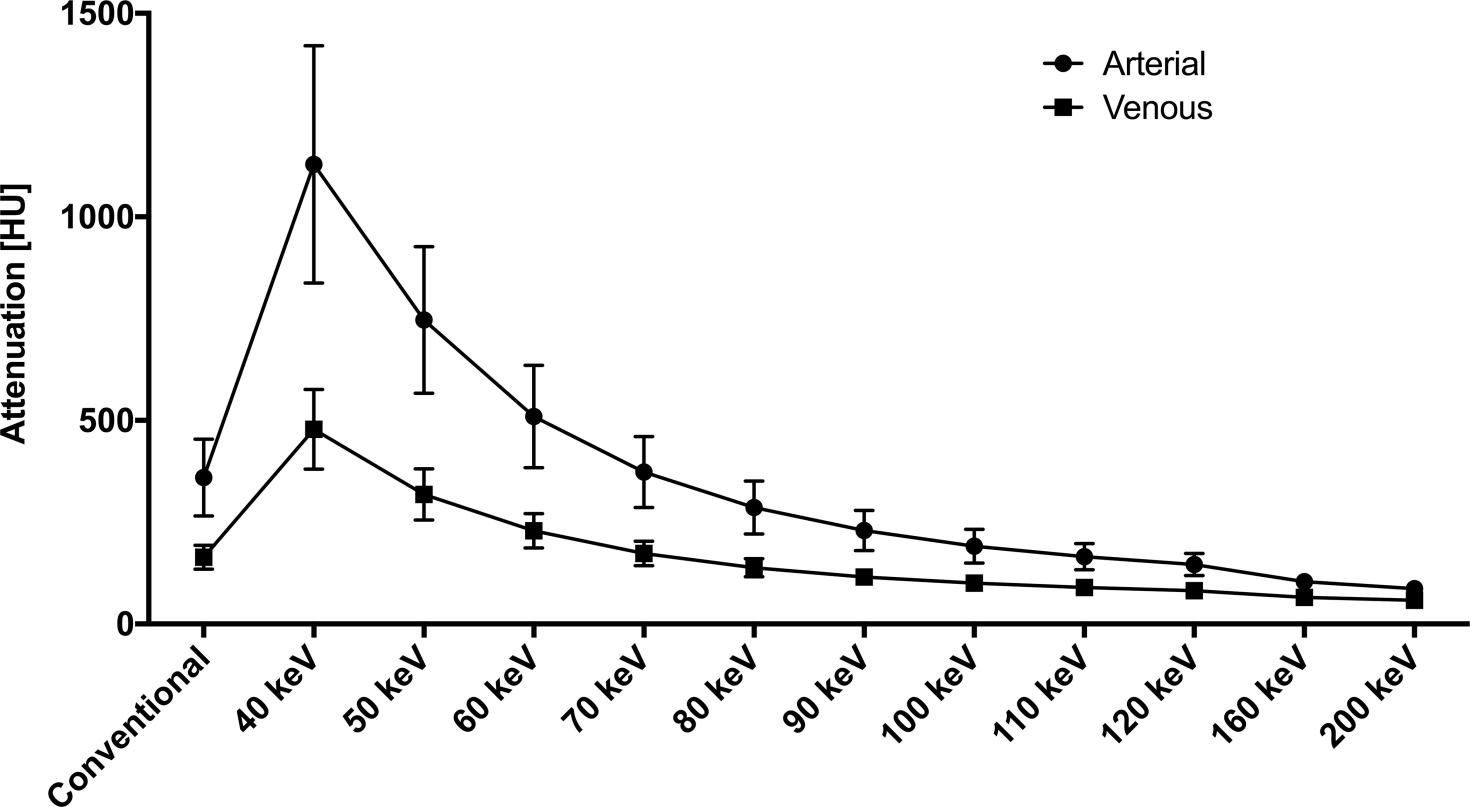
Attenuation values assessed in the suprarenal aorta in arterial and venous contrast phase imaging. The highest values are found for MonoE at 40 keV reconstruction level, respectively. (values: mean ± SD)

Vascular attenuation in MonoE showed a stepwise increase from high to low keV levels in arterial and venous phase imaging. Representatively, all values in the suprarenal aorta are shown in Fig 1. In arterial phase, all evaluated vessels showed higher attenuation values at 40, 50, and 60 keV MonoE reconstruction levels (p ≥ 0.001) compared to the corresponding conventional reconstructions. At 70 keV, attenuation values were comparable (p > 0.99) and reconstruction levels of ≥ 90 keV showed lower attenuation values (p < 0.001) than the conventional images.

Analogous, in venous phase imaging, all evaluated vessels showed higher attenuation values at 40, 50, and 60 keV MonoE reconstruction levels (p ≥ 0.001) compared to the corresponding conventional images. The mean percentage increase of the attenuation values for the 40, 50, and 60 keV MonoE reconstructions were: 190, 91, and 37%, respectively. The MonoE at 70 keV and the corresponding conventional reconstruction yielded comparable attenuation values (p ≥ 0.98). Attenuation values of MonoE gradually decreased and showed lower attenuation values at ≥ 90 keV compared to the conventional images (p < 0.001). Absolute attenuation values derived from MonoE 40 keV in venous contrast phase showed higher values than derived from conventional images in arterial phase (415.5 ± 44.36 HU vs. 318.9 ± 29.65 HU; p < 0.001).

Compared to conventional images, absolute attenuation in MonoE of the liver, pancreas and kidneys showed significant higher values at 40, 50, and 60 keV (all ≤0.001).

#### Image noise

**Fig 2:**
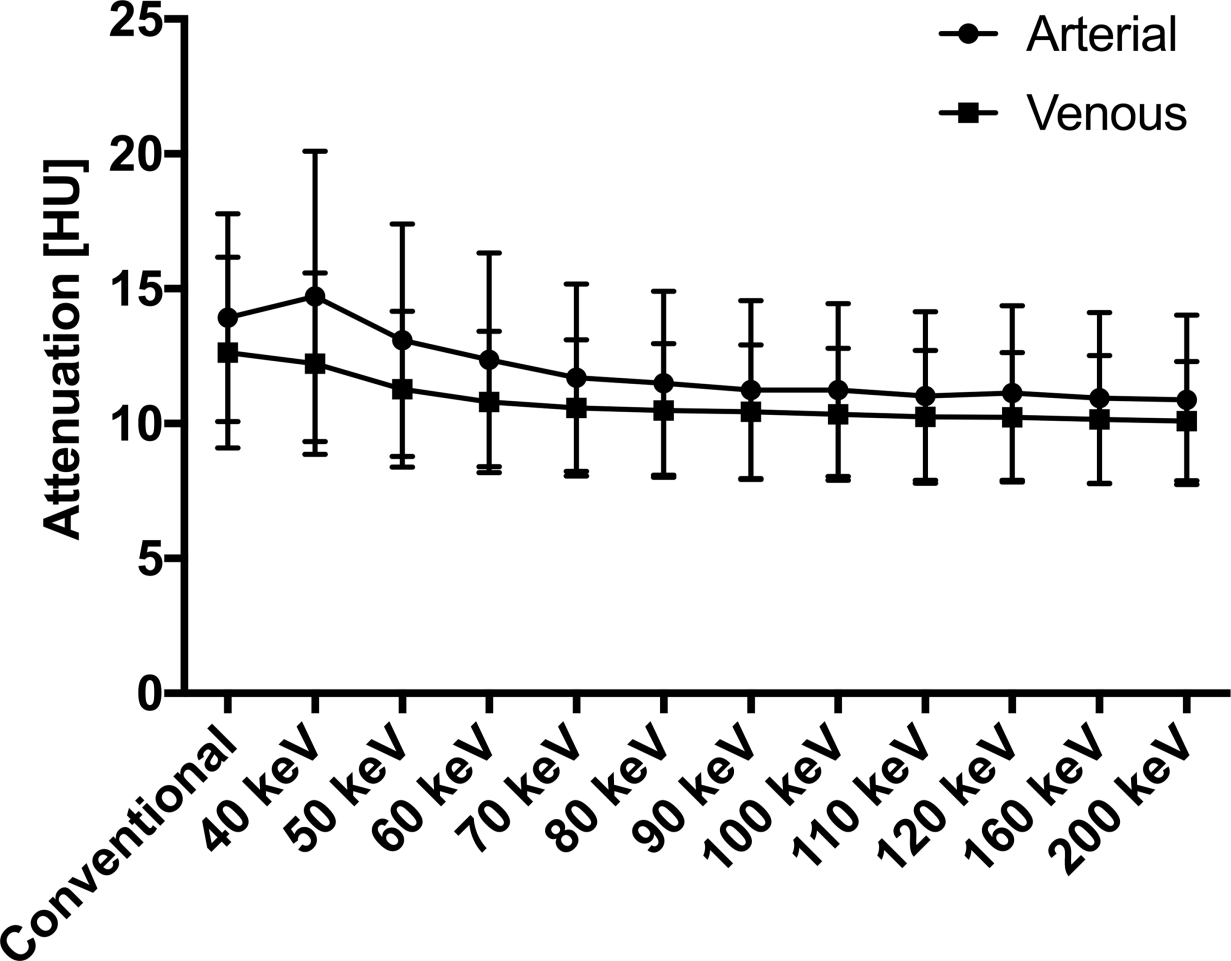
Image noise for the whole energy spectrum from 40 to 200 keV in both contrast phases (values: Mean ± SD). Note that image noise values are not significantly increased in the low keV MonoE reconstruction levels compared to the high keV reconstructions.

Image noise levels in arterial and venous phase MonoE and conventional datasets are shown in Fig 2. In arterial contrast phase, even the 40 keV MonoE reconstructions showed slightly, yet not significant lower image noise compared to the corresponding conventional reconstruction (12.2 ± 3.4 HU vs. 12.6 ± 3.5 HU; p > 0.99). Image noise gradually decreased below the level of the conventional datasets at reconstruction levels > 40 keV reaching statistical significance at 60 keV.

In venous contrast phase, image noise in the 40 keV MonoE reconstruction exceeded slightly but not significantly the image noise of the corresponding conventional dataset (14.7 ± 5.4 HU vs. 13.3 ± 3.9 HU; p > 0.99), whereas reconstruction levels > 40 keV showed a gradual decrease of image noise levels below that of the conventional reconstructions, reaching statistical significance at 80 keV.

Noise levels were significantly higher for MonoE of the liver and the kidneys at 40 keV reconstruction compared to conventional (p ≤ 0.02). Noise levels of the liver and the kidneys were statistically inferior at levels of ≥ 80 keV (p ≤ 0.005). Noise levels of the pancreas were statistically inferior at levels of ≥ 70 keV (p ≤ 0.008).

#### Contrast to noise (CNR)

All CNR values are shown in detail in Table 1. In general, CNR values of MonoE gradually decreased from low to high keV levels. In arterial and venous phase, the 40, 50, and 60 keV MonoE reconstructions yielded superior CNR values than the conventional reconstructions with regards to all evaluated vessels (p ≤ 0.001). Even the CNR value of the venous phase 40 keV MonoE reconstruction surpassed the CNR value of the corresponding arterial phase conventional reconstruction. The mean percentage increase of the CNR values for the 40, 50, and 60 keV MonoE reconstructions were: 248, 142, and 73%, respectively. CNR values at 80 keV were equivalent to the corresponding conventional images for all evaluated vessels (p > 0.99). In contrast, CNR values of MonoE reconstructed with ≥ 110 keV were inferior to the CNR values yielded by conventional images (p ≤ 0.03).

**Table 1:** Contrast-to-noise (CNR) values (mean ± SD) of conventional and virtual mono-energetic images (all keV levels) split for all anatomic regions and separately given for arterial and venous phase.

CNR	CM-Phase	120 KV	40 keV	*p-value*	50 keV	*p-value*	60 keV	*p-value*	70 keV	*p-value*	80 keV	*p-value*	90 keV	*p-value*	100 keV	*p-value*	110 keV	*p-value*	120 keV	*p-value*	160 keV	*p-value*	200 keV	*p-value*
		Conventional																						
Suprarenal aorta	art.	27.3 ± 12.7	98.3 ± 46.9	**<0.001**	68.3 ± 31.9	**<0.001**	47.4 ± 22.1	**<0.001**	33.6 ± 15.6	0.86	24.8 ± 10.7	>0.99	18.8 ± 8.2	0.49	14.9 ± 6.7	**0.04**	12.4 ± 5.5	**0.003**	10.3 ± 4.6	**<0.001**	5.9 ± 2.8	**<0.001**	4.1 ± 1.7	**<0.001**
	ven.	9.0 ± 4.4	32.7 ± 18.3	**<0.001**	22.9 ± 12.8	**<0.001**	16.3 ± 8.6	**<0.001**	11.7 ± 6.1	0.77	8.7 ± 4.8	>0.99	6.5 ± 3.5	0.85	5.0 ± 2.8	0.18	4.0 ± 2.5	**0.03**	3.2 ± 2.0	**0.004**	1.7 ± 1.3	**<0.001**	0.9 ± 1.2	**<0.001**
Infrarenal aorta	art.	27.0 ± 13.5	96.1 ± 48.7	**<0.001**	67.2 ± 33.4	**<0.001**	46.8 ± 23.1	**<0.001**	33.6 ± 16.2	0.87	24.8 ± 11.3	>0.99	19.1 ± 8.7	0.67	15.3 ± 6.9	0.10	12.6 ± 5.7	**0.01**	10.6 ± 4.8	**0.002**	6.2 ± 3.1	**<0.001**	4.3 ± 1.8	**<0.001**
	ven.	9.1 ± 4.6	33.2 ± 19.1	**<0.001**	23.3 ± 13.4	**<0.001**	16.5 + 8.9	**<0.001**	11.6 + 6.3	0.88	8.7 ± 4.9	>0.99	6.6 ± 3.6	0.88	4.9 ± 2.8	0.20	4.1 + 2.5	**0.04**	3.2 ± 1.9	**0.005**	1.6 ± 1.2	**<0.001**	0.9 ± 1.0	**<0.001**
Celiac artery	art.	24.4 ± 10.4	82.8 ± 35.7	**<0.001**	57.3 ± 24.4	**<0.001**	40.7 ± 16.6	**<0.001**	29.3 ± 12.3	0.85	21.8 ± 8.1	>0.99	16.9 ± 6.2	0.29	13.7 ± 5.3	**0.01**	11.3 ± 4.4	**<0.001**	9.6 ± 3.7	**<0.001**	6.0 ± 2.6	**<0.001**	4.5 ± 2.1	**<0.001**
	ven.	8.2 ± 4.6	28.4 ± 17.2	**<0.001**	20.2 ± 12.3	**<0.001**	14.2 ± 8.1	**<0.001**	10.1 ± 6.0	0.96	7.7 ± 4.7	>0.99	5.9 ± 3.6	0.90	4.5 ± 2.9	0.27	3.5 ± 3.4	**0.04**	3.0 ± 2.3	**0.01**	1.6 ± 1.7	**<0.001**	1.1 ± 1.4	**<0.001**
Common hepatic artery	art.	21.0 ± 10.1	80.2 ± 33.5	**<0.001**	47.1 ± 22.8	**<0.001**	40.1 ± 16.0	**<0.001**	24.1 ± 12.1	>0.99	18.2 ± 9.6	>0.99	14.4 ± 7.6	0.50	12.0 ± 6.8	0.09	10.0 ± 5.8	**0.009**	8.8 ± 5.5	**0.002**	5.9 ± 4.1	**<0.001**	4.3 ± 1.9	**<0.001**
	ven.	7.1 ± 4.1	24.7 ± 14.2	**<0.001**	17.3 ± 9.7	**<0.001**	12.1 ± 6.7	**<0.001**	8.3 ± 4.6	>0.99	6.3 ± 3.9	>0.99	4.8 ± 3.0	0.65	3.6 ± 2.5	0.09	2.8 ± 2.2	**0.008**	2.3 ± 2.0	**0.001**	1.1 ± 1.7	**<0.001**	0.6 ± 1.5	**<0.001**
Superior mesenteric artery	art.	24.0 ± 10.3	79.3 ± 36.2	**<0.001**	56.1 ± 25.2	**<0.001**	39.1 ± 16.2	**<0.001**	28.2 ± 11.7	0.94	20.9 ± 7.8	>0.99	16.2 ± 6.2	0.23	13.1 ± 5.0	**0.009**	10.9 ± 4.3	**<0.001**	9.3 ± 3.6	**<0.001**	5.7 ± 2.8	**<0.001**	4.3 ± 2.3	**<0.001**
	ven.	7.8 ± 3.8	27.8 ± 15.6	**<0.001**	19.5 ± 10.8	**<0.001**	13.8 ± 7.3	**<0.001**	9.5 ± 5.0	0.97	7.2 ± 3.7	>0.99	5.4 ± 2.8	0.71	4.1 ± 2.3	0.09	3.3 ± 1.9	**0.010**	2.6 ± 1.6	**0.001**	1.2 ± 1.2	**<0.001**	0.6 ± 1.0	**<0.001**
Splenic artery	art.	22.3 ± 10.7	71.2 ± 30.1	**<0.001**	49.8 ± 21.3	**<0.001**	35.9 ± 14.9	**<0.001**	25.8 ± 11.1	0.98	19.4 ± 7.2	>0.99	15.3 ± 6.0	0.27	12.4 ± 5.1	**0.01**	10.6 ± 4.4	**<0.001**	9.0 ± 3.8	**<0.001**	6.0 ± 3.0	**<0.001**	4.9 ± 2.7	**<0.001**
	ven.	7.4 ± 4.1	26.5 ± 15.9	**<0.001**	18.6 ± 11.1	**<0.001**	13.0 ± 7.4	**<0.001**	9.2 ± 5.5	0.96	7.0 ± 4.4	>0.99	5.2 ± 3.3	0.83	4.0 ± 2.7	0.25	3.2 ± 2.6	0.05	2.7 ± 2.2	**0.01**	1.4 ± 1.7	**<0.001**	0.9 ± 1.5	**<0.001**
Left renal artery	art.	21.3 ± 11.3	68. 4 ± 35.7	**<0.001**	47.6 ± 24.1	**<0.001**	34.9 ± 16.9	**<0.001**	25.4 ± 12.5	0.97	19.3 ± 8.7	>0.99	15.4 ± 7.7	0.71	12.7 ± 6.0	0.16	10.9 ± 5.6	**0.03**	9.3 ± 5.1	**0.004**	6.6 ± 4.0	**<0.001**	5.2 ± 2.6	**<0.001**
	ven.	6.8 ± 3.9	24.6 ± 15.1	**<0.001**	17.5 ± 10.8	**<0.001**	12.4 ± 7.2	**<0.001**	8.6 ± 5.2	0.95	6.4 ± 4.2	>0.99	4.8 ± 3.1	0.87	3.7 ± 2.6	0.28	2.9 ± 2.4	0.05	2.4 ± 2.1	**0.01**	1.1 ± 1.5	**<0.001**	0.6 ± 1.4	**<0.001**
Right renal artery	art.	20.5 ± 8.7	63.7 ± 29.5	**<0.001**	45.0 ± 19.5	**<0.001**	33.0 ± 14.2	**<0.001**	24.0 ± 10.7	0.97	18.2 ± 6.4	>0.99	14.4 ± 5.2	0.43	12.1 ± 4.4	0.06	10.4 ± 3.8	**0.006**	9.0 ± 3.3	**<0.001**	6.6 ± 3.2	**<0.001**	5.1 ± 2.3	**<0.001**
	ven.	6.8 ± 3.7	24.6 ± 13.7	**<0.001**	17.1 ± 10.0	**<0.001**	12.1 ± 6.9	**<0.001**	8.3 ± 4.8	0.98	6.6 ± 4.1	>0.99	4.9 ± 3.2	0.89	3.8 ± 2.7	0.29	3.3 ± 2.5	0.07	2.5 ± 2.2	**0.01**	1.4 ± 1.6	**<0.001**	0.8 ± 1.6	**<0.001**
Portal vein	ven.	9.0 ± 3.9	33.8 ± 18.8	**<0.001**	23.7 ± 12.8	**<0.001**	16.7 ± 8.3	**<0.001**	11.7 ± 5.9	0.74	8.7 ± 4.3	>0.99	6.6 ± 3.2	0.90	5.1 ± 2.5	0.23	4.0 ± 2.2	**0.003**	3.3 ± 1.8	**0.005**	1.7 ± 1.2	**<0.001**	0.9 ± 0.9	**<0.001**
Liver	ven.	4.1 ± 3.1	13.1 ± 9.3	**<0.001**	10.0 ± 7.4	**<0.001**	6.7 ± 4.8	0.06	5.0 ± 3.7	>0.99	4.1 ± 3.1	>0.99	3.2 ± 2.5	>0.99	2.7 ± 2.3	0.90	2.4 ± 2.1	0.62	2.0 ± 2.0	0.34	1.5 ± 1.7	0.08	1.3 ± 1.6	**0.03**
Pancreas	ven.	2.9 ± 2.1	12.0 ± 7.9	**<0.001**	8.3 ± 5.7	**<0.001**	5.5 ± 3.9	**0.004**	3.4 ± 2.7	>0.99	2.4 ± 2.2	>0.99	1.5 ± 1.6	0.59	0.9 ± 1.4	0.10	0.4 ± 1.2	**0.009**	0.1 ± 1.2	**0.001**	-0.5 ± 1.0	**<0.001**	-0.8 ± 1.1	**<0.001**
Kidney	ven.	11.0 ± 5.7	39.9 ± 24.2	**<0.001**	27.9 ± 17.2	**<0.001**	19.6 ± 11.3	**<0.001**	13.6 ± 8.1	0.73	10.2 ± 6.3	>0.99	7.6 ± 4.5	0.38	5.7 ± 3.5	**0.007**	4.4 ± 2.9	**<0.001**	3.5 ± 2.6	**<0.001**	1.5 ± 1.5	**<0.001**	0.7 ± 1.1	**<0.001**

Bold indicating statistical significance; SNR = signal-to-noise ratio, CM-Phase = contrast medium phase, art. = arterial, ven. = venous

In solid organs, CNR values showed a similar behavior with statistical significant higher values in MonoE at 40 and 50 keV of the liver and the kidneys and at 40, 50, and 60 keV in MonoE of the pancreas. Statistical significant lower values were found for MonoE ≥ 100 keV for the kidneys, ≤ 110 keV for the pancreas and 200 keV for the liver compared to conventional reconstructions.

#### Signal to noise (SNR)

All SNR values are shown in detail in Table 2. For both contrast phases the 40 and 50 keV MonoE reconstruction yielded superior SNR in all evaluated vessels than the conventional reconstruction (p ≤ 0.04). SNR levels in MonoE at 40 and 50 keV reconstruction in venous phase were superior compared to conventional images in arterial phase imaging (25.3 ± 7.7 and 19.6 ± 4.0 vs. 17.9 ± 4.6). At 60 keV, SNR was higher in the supra- and infrarenal aorta as well as the CHA, SMA, SA and left RA in arterial and venous phase compared to the corresponding conventional reconstruction (p ≤ 0.04). SNR declined from low to high keV MonoE reconstruction below the SNR of the corresponding conventional images, reaching statistical significance at 160 keV in all vessels except of the SA in the arterial phase (p = 0.05), the coeliac trunk (p = 0.30) and the SMA (p = 0.11) in the venous phase. At 200 keV reconstruction level the SNR of all evaluated vessels dropped below the SNR yielded by the conventional reconstructions in either contrast phase. The mean percentage increase of the SNR values for the 40, 50, 60, and 70 keV MonoE reconstructions were: 129, 81, 42 and 12%, respectively.

**Table 2:** Signal-to-noise (SNR) values (mean ± SD) of conventional and virtual mono-energetic images (all keV levels) split for all anatomic regions and separately given for arterial and venous phase.

SNR	CM-Phase	120 KV	40 keV	*p-value*	50 keV	*p-value*	60 keV	*p-value*	70 keV	*p-value*	80 keV	*p-value*	90 keV	*p-value*	100 keV	*p-value*	110 keV	*p-value*	120 keV	*p-value*	160 keV	*p-value*	200 keV	*p-value*
		Conventional																						
Suprarenal aorta	art.	25.8 ± 10.5	78.9 ± 29.9	**<0.001**	57.6 ± 22.1	**<0.001**	40.6 ± 17.8	**<0.001**	31.1 ± 12.6	0.67	24.4 ± 9.8	>0.99	19.51 ± 7.7	0.39	16.4 ±6.4	**0.02**	14.1 ± 5.1	**<0.001**	12.6 ± 4.4	**<0.001**	8.8 ± 3.0	**<0.001**	7.3 ± 2.2	**<0.001**
	ven.	11.7 ± 4.6	40.2 ± 15.9	**<0.001**	27.4 ± 11.2	**<0.001**	20.3 ± 7.9	**<0.001**	15.5 ± 6.0	0.16	12.6 ± 4.6	>0.99	10.4 ± 3.8	>0.99	9.1 ± 3.1	0.66	8.0 ± 2.8	0.17	7.3 ± 2.5	**0.04**	5.8 ± 1.9	**<0.001**	5.2 ± 1.6	**<0.001**
Infrarenal aorta	art.	23.7 ± 11.0	72.6 ± 31.6	**<0.001**	52.2 ± 21.5	**<0.001**	37.2 ± 14.6	**<0.001**	28.3 ± 11.1	0.83	22.0 ± 8.2	>0.99	17.8 ± 6.7	0.49	15.0 ± 5.5	**0.04**	12.9 ± 4.7	**0.002**	11.5 ± 4.2	**<0.001**	8.2 ± 2.9	**<0.001**	6.9 ± 2.1	**<0.001**
	ven.	10.2 ± 3.6	34.6 ± 13.1	**<0.001**	24.3 ± 8.8	**<0.001**	17.8 ± 6.2	**<0.001**	13.6 ± 4.9	0.06	10.9 ± 3.6	>0.99	9.2 ± 3.0	>0.99	7.9 ± 2.5	0.57	7.2 ± 2.3	0.16	6.5 + 1.9	**0.02**	5.1 ± 1.4	**<0.001**	4.5 ± 1.3	**<0.001**
Celiac artery	art.	16.8 ± 10.5	26.7 ± 19.8	**<0.001**	22.9 ± 14.6	**0.04**	19.5 ± 10.6	0.95	17.1 ± 8.9	>0.99	14.8 ± 7.1	>0.99	13.1 ± 6.2	0.71	11.8 ± 5.4	0.25	10.6 ± 4.8	0.05	9.8 ± 4.5	**0.01**	7.9 ± 3.5	**<0.001**	7.6 ± 4.8	**<0.001**
	ven.	10.1 ± 5.7	23.8 + 18.8	**<0.001**	19.0 ± 12.8	**<0.001**	15.4 ± 9.4	0.05	12.5 + 7.6	0.94	10.9 + 6.2	>0.99	9.6 ± 5.3	>0.99	8.7 ± 4.9	>0.99	7.6 + 4.5	0.91	7.2 ± 3.8	0.8	6.0 ± 3.2	0.3	5.4 ± 2.9	0.13
Common hepatic artery	art.	13.3 ± 8.1	32.0 ± 17.8	**<0.001**	23.0 + 9.7	**<0.001**	19.1 ± 8.6	**0.04**	12.7 + 7.8	>0.99	10.4 +5.0	0.85	9.1 ± 4.6	0.3	7.1 ± 2.2	**0.02**	6.8 + 2.1	**0.009**	6.5 + 2.1	**0.003**	5.9 ± 2.3	**<0.001**	5.5 ± 1.4	**<0.001**
	ven.	9.7 ± 5.0	20.1 ± 11.7	**<0.001**	16.4 ± 8.2	**<0.001**	13.6 ± 6.5	**0.02**	9.1 ± 3.6	>0.99	7.5 ± 2.1	0.79	6.5 ± 1.8	0.2	6.0 ± 1.6	0.06	5.6 ± 1.6	**0.02**	5.2 ± 1.6	**0.006**	4.4 ± 1.5	**<0.001**	4.2 ± 1.5	**<0.001**
Superior mesenteric artery	art.	18.4 ± 8.0	34.1 ± 23.7	**<0.001**	28.5 ± 16.7	**0.009**	23.5 ± 12.4	0.74	17.7 ± 7.8	>0.99	15.0 ± 6.0	0.98	12.6 ± 4.3	0.58	10.9 ± 3.4	0.2	9.7 ± 2.9	0.06	9.0 ± 2.4	**0.02**	7.3 ± 1.8	**0.002**	6.1 ± 1.4	**<0.001**
	ven.	10.4 ± 5.6	23.3 ± 18.3	**<0.001**	18.5 ± 12.5	**<0.001**	15.8 ± 9.2	0.22	11.8 ± 6.1	>0.99	10.1 ± 4.9	>0.99	8.9 ± 4.4	>0.99	8.0 ± 3.8	0.96	7.2 ± 3.3	0.75	6.7 ± 3.0	0.53	5.5 ± 2.4	0.11	5.2 ± 2.4	0.07
Splenic artery	art.	16.0 ± 8.6	26.9 ± 14.0	**<0.001**	24.1 ± 11.1	**0.02**	19.4 ± 8.2	0.94	18.2 ± 8.8	>0.99	15.4 ± 7.8	>0.99	13.6 ± 6.6	>0.99	12.3 ± 6.3	0.91	11.1 ± 5.7	0.61	10.4 ± 5.2	0.4	8.4 ± 4.0	0.05	7.4 ± 3.2	**0.01**
	ven.	9.4 ± 3.7	20.1 ± 11.7	**<0.001**	16.5 ± 8.1	**<0.001**	13.7 ± 6.0	**0.002**	11.3 ± 4.5	0.78	9.8 ± 3.6	>0.99	8.5 ± 3.0	>0.99	7.8 ± 2.6	0.89	7.1 ± 2.3	0.46	6.5 ± 2.1	0.17	5.5 ± 1.7	**0.008**	5.2 ± 1.7	**0.002**
Left renal artery	art.	13.7 ± 5.4	20.5 ± 10.8	**<0.001**	18.9 ± 8.7	**0.01**	16.4 ± 7.4	0.75	14.3 ± 5.6	>0.99	12.4 ± 5.2	>0.99	11.7 ± 4.2	0.95	10.8 ± 3.8	0.64	9.9 ± 3.3	0.21	9.0 ± 3.1	**0.04**	7.9 ± 2.5	**0.002**	7.2 ± 2.2	**<0.001**
	ven.	9.5 ± 4.9	20.6 ± 12.5	**<0.001**	17.4 ± 10.7	**<0.001**	14.4 ± 9.2	**0.02**	10.8 ± 6.3	>0.99	9.9 ± 5.5	>0.99	7.9 ± 3.3	>0.99	7.2 ± 3.1	0.88	6.6 ± 2.9	0.61	6.2 ± 2.6	0.43	5.2 ± 2.1	0.09	4.6 ± 1.9	**0.03**
Right renal artery	art.	15.2 ± 6.6	23.8 ± 13.5	**<0.001**	21.1 ± 8.7	**0.03**	18.5 ± 7.4	0.77	15.5 ± 5.1	>0.99	13.7 ± 4.2	>0.99	12.3 ± 3.8	0.89	11.2 ± 3.5	0.51	10.4 ± 3.1	0.2	9.9 ± 3.1	0.1	8.3 ± 2.9	**0.006**	7.4 ± 2.3	**<0.001**
	ven.	9.4 ± 5.3	19.8 ± 12.1	**<0.001**	17.4 ± 11.6	**<0.001**	13.9 ± 8.4	**0.2**	11.5 + 6.6	0.98	9.6 ± 5.4	>0.99	8.5 ± 4.4	>0.99	7.7 ± 4.0	>0.99	7.1 ± 3.7	0.96	6.5 ± 3.3	0.85	5.5 ± 2.8	0.45	5.0 ± 2.4	0.26
Portal vein	ven.	10.7 ± 3.4	33.3 ± 14.1	**<0.001**	25.5 ± 9.2	**<0.001**	18.6 ± 6.6	**<0.001**	14.4 ± 5.1	0.06	11.7 ± 3.8	>0.99	9.9 ± 3.1	>0.99	8.6 ± 2.7	0.8	7.5 ± 2.4	0.18	7.0 ±2.1	0.05	5.6 ± 1.6	**<0.001**	4.9 ± 1.4	**<0.001**
Liver	ven.	7.7 ± 3.2	16.9 ± 10.3	**<0.001**	14.3 ± 7.9	**<0.001**	11.6 ± 5.7	**0.002**	10.0 ± 4.5	0.37	9.0 ± 3.9	0.95	8.3 ± 3.4	>0.99	7.7 ± 3.0	>0.99	7.3 ± 2.9	>0.99	7.1 ± 2.7	>0.99	6.4 ± 2.4	0.98	6.2 ± 2.2	0.94
Pancreas	ven.	5.4 ± 2.3	13.7 ± 6.5	**<0.001**	10.7 ± 4.8	**<0.001**	8.4 ± 3.6	**<0.001**	7.0 ± 2.8	0.23	5.9 ± 2.2	>0.99	5.3 ± 1.9	>0.99	4.9 ± 1.6	>0.99	4.4 ± 1.5	0.84	4.2 ± 1.5	0.55	3.6 ± 1.2	0.09	3.4 ± 1.1	**0.03**
Kidney	ven.	14.1 ± 7.0	38.3 ± 25.4	**<0.001**	29.9 ± 17.8	**<0.001**	23.3 ± 13.2	**<0.001**	18.3 ± 9.9	0.29	15.2 ± 7.6	>0.99	12.6 ± 6.1	>0.99	10.8 ± 5.1	0.66	9.6 ± 4.5	0.19	8.8 ± 4.0	0.05	6.9 ± 3.0	**<0.001**	6.0 ± 2.4	**<0.001**

Bold indicating statistical significance; SNR = signal-to-noise ratio, CM-Phase = contrast medium phase, art. = arterial, ven. = venous

SNR values of solid organs showed also a stepwise decrease from low to high keV reconstruction levels. Values were statically superior in MonoE at 40, 50, and 60 keV reconstructions, compared to conventional images (p ≤ 0.009). SNR values were statically inferior for the kidneys at levels ≥ 160 keV, and 200 keV for the pancreas, compared to conventional images. In contrast, no significant differences were found for the liver at high keV reconstruction levels compared to conventional.

**Fig 3:**
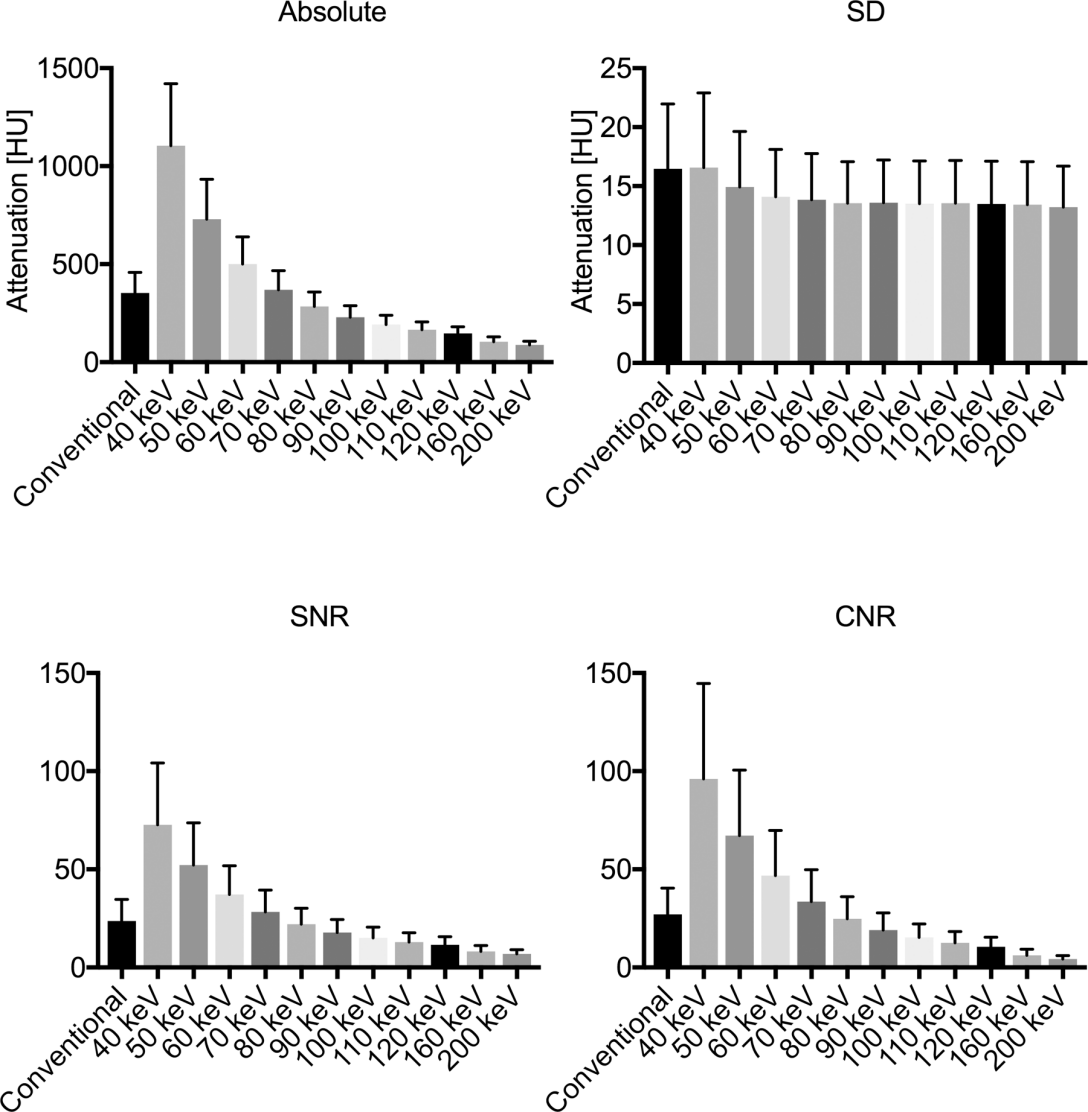
Absolute attenuation, standard deviation (SD), SNR and CNR in the infrarenal aorta in arterial contrast phase imaging for conventional and MonoE reconstructions. Due to the substantial increase of absolute attenuation and constantly low SDs, SNR and CNR values are markedly elevated at low keV reconstruction levels (Values: Mean ± SD).

A complete representative data set in arterial phase measured in the infrarenal aorta is shown in Fig 3.

#### Qualitative image analysis

**Fig 4:**
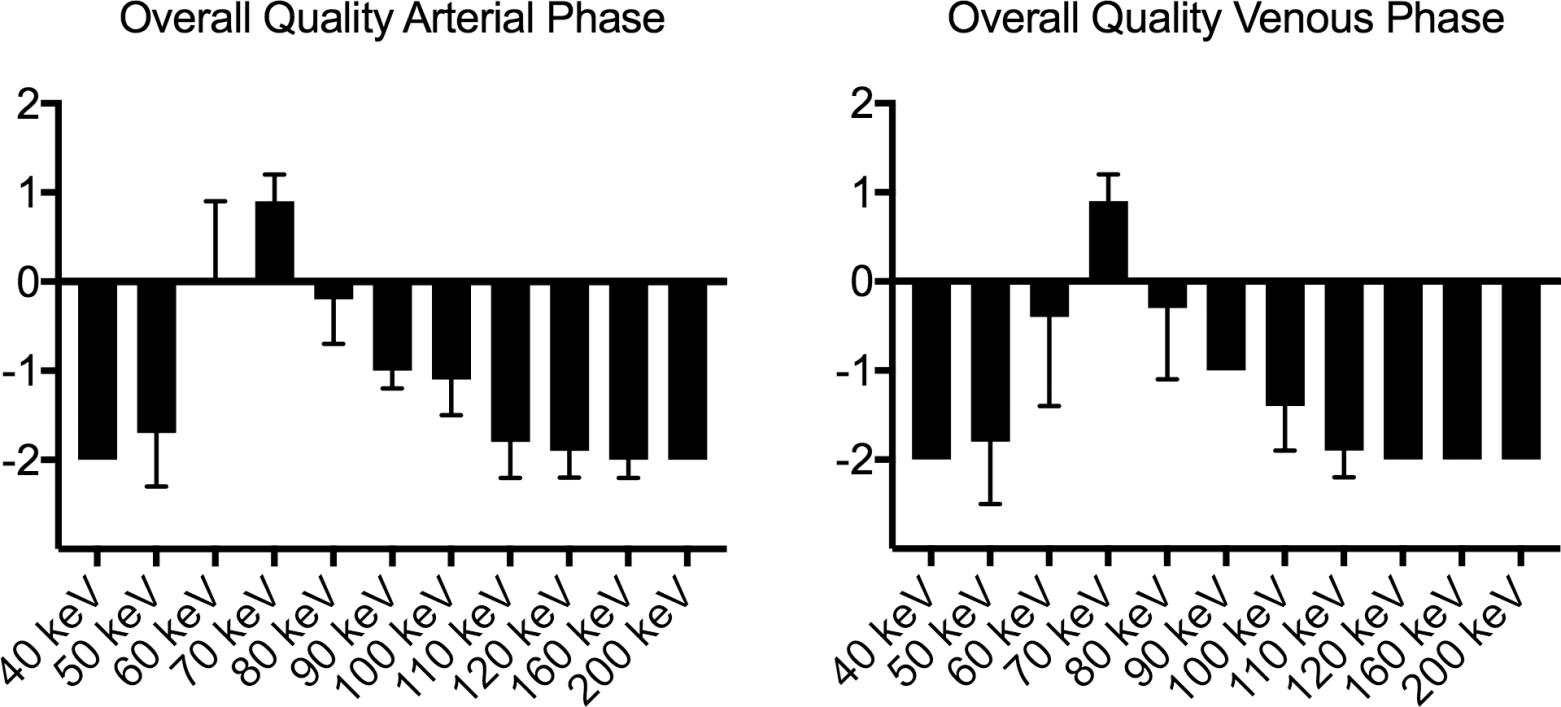
Waterfall plot showing overall image quality rating in arterial and venous contrast phase with standard abdominal window settings where 0 corresponds to the reference standard (conventional imaging). Best image quality was rated for MonoE at 70 keV in arterial and 80 keV in venous phase.

**Fig 5:**
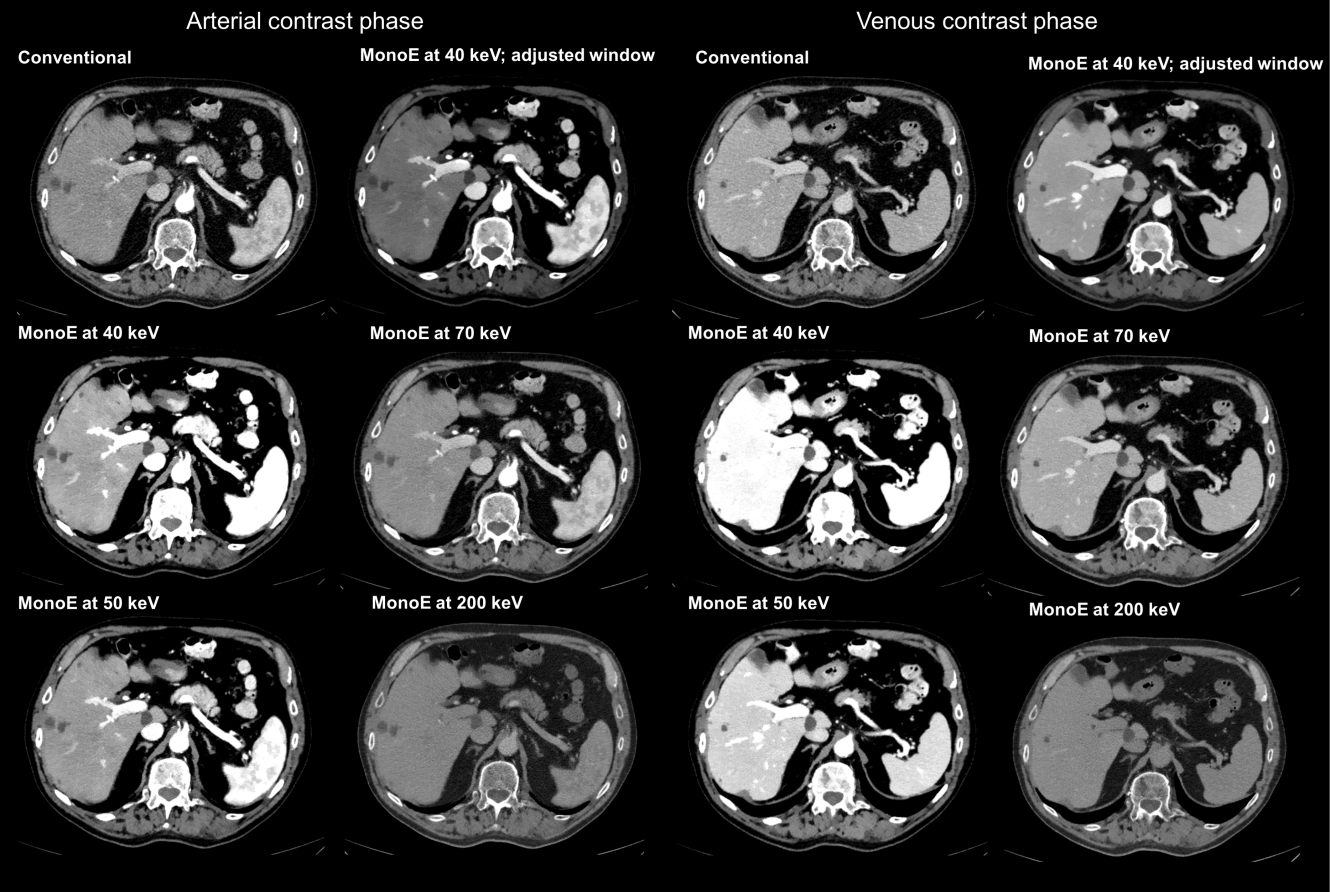
SDCT images of an 83-year old patient in arterial and venous contrast phase. For a more realistic comparability window settings were kept constant (center 60 HU; width 360 HU), except for the 40 keV MonoE reconstructions with adjusted window settings. Compared to conventional images MonoE at low keV reconstruction levels showed increased attenuation and contrast without excessive noise in both contrast phases. If window levels were adjustable MonoE at 40 keV was rated best for overall image quality, whereas reconstruction levels of 70 keV were rated best and superior to conventional images if readers were not allowed to adjust window settings. Note extensive increase in parenchymal attenuation in venous contrast phase in MonoE at 40 keV reconstruction level with standard windowing presets.

Scores of the qualitative image analysis were dependent on adjustability of the window settings. Overall image quality for arterial and venous imaging is shown in Fig 4 and all inter-reader data is given in Table 3. Without adjustment of the standard window setting the 70 keV MonoE reconstruction yielded the best overall image quality in arterial and venous phase imaging. Inter-reader agreement was substantial (both contrast phases: κ = 0771.). Conversely, when window adjustment was explicitly allowed the 40 keV MonoE reconstructions yielded the best subjective image quality in arterial and venous phase imaging. Inter-reader agreement was excellent (κ n/a). Representative images are shown in Fig 5.

**Table 3:** Qualitative image analysis (subjective image contrast, subjective visualization of details, subjective overall image quality) of the virtual mono-energetic images (all keV levels) given as means ± SD split for arterial and venous phase.

MonoE	CM-Phase	40 keV	*Kappa*	50 keV	*Kappa*	60 keV	*Kappa*	70 keV	*Kappa*	80 keV	*Kappa*	90 keV	*Kappa*	100 keV	*Kappa*	110 keV	*Kappa*	120 keV	*Kappa*	160 keV	*Kappa*	200 keV	*Kappa*
Contrast	art.	2.0 ± 0	n/a	1.9 ± 0.2	0.642	1.0 ± 0.4	0.779	0.1 ± 0.3	0.771	-0.6 ± 0.5	0.894	-0.9 ± 0.3	0.642	-1.3 ± 0.5	0.872	-1.8 ± 0.4	0.826	-1.9 ± 0.2	1	-1.9± 0.3	0.642	-2.0 ± 0	n/a
	ven.	2.0 ± 0	n/a	1.9 ± 0.2	1	1.5 ± 0.5	0.789	0.6 ± 0.5	0.890	-0.3 ± 0.4	0.732	-1.0 ± 0.2	1	-1.6 ± 0.5	0.776	-1.9 ± 0.3	0.642	-2.0 ± 0	n/a	-2.0 ± 0	n/a	-2.0 ± 0	n/a
Visualization of details	art.	-2.0 ± 0	n/a	-1.4 + 0.8	0.904	-0.1 ± 0.9	0.921	0.4 ± 0.5	0.894	0.1 ± 0.4	0.776	-0.5 + 0.5	0.895	-1.0 ± 0.4	0.779	-1.2 ± 0.4	0.689	-1.8 ± 0.4	0.826	-1.9 ± 0.2	1	-2.0 ± 0.2	n/a
	ven.	-2.0 ± 0	n/a	-1.8 ± 0.6	0.808	-0.1 ± 1.0	0.906	0.8 ± 0.4	0.855	0.2 ± 0.5	0.877	-0.4 ± 0.6	0.904	-1.0 ± 0.2	1	-1.6 ± 0.5	0.890	-2.0 ± 0.2	1	-2.0 ± 0	n/a	-2.0 ± 0	n/a
Quality	art.	-2.0 ± 0	n/a	-1.7 ± 0.6	1	0.0 ± 0.9	0.917	0.9 ± 0.3	0.771	-0.2 ± 0.5	0.891	-1.0 ± 0.2	1	-1.1 ± 0.4	0.776	-1.8 ± 0.4	1	-1.9 ± 0.3	0.642	-2.0 ± 0.2	1	-2.0 ± 0	n/a
	ven.	-2.0 ± 0	n/a	-1.8 ± 0.7	1	-0.4 ± 1.0	0.814	0.9 ± 0.3	0.771	-0.3 ± 0.8	0.829	-1.0 ± 0	n/a	-1.4 ± 0.5	0.894	-1.9 ± 0.3	0.642	-2.0 ± 0	n/a	-2.0 ± 0	n/a	-2.0 ± 0	n/a

MonoE = mono-energetic imaging, CM-Phase = contrast medium phase, art. = arterial, ven. = venous, n/a = not applicable

## Discussion

To the authors`knowledge, this study is the first intra-individual comparison of quantitative and qualitative image parameters for MonoE and conventional reconstructions in abdominal arterial and venous phase contrast-enhanced scans acquired on a novel SDCT scanner. We found that MonoE reconstructions at lower keV levels yielded significant higher attenuation values compared to conventional reconstructions or higher keV levels. Most interestingly however, our study revealed a consistently low image noise across the energy spectrum even at the lowest keV levels. As a synergistic effect to the attenuation boost close to the iodine k-edge (33 keV), the low image noise yields increased CNR and SNR at 40 and 50 keV in both, arterial and venous contrast phase imaging. The CNR of venous MonoE reconstruction using 40 keV and in terms of SNR also with 50 keV even surpassed conventional images. Our findings are in contrast to older studies using first generation dual energy systems showing the CNR and SNR peaks around 70 keV [5, 8], but in good accordance with recently published data using third-generation dual-source systems using an advanced noise-optimized virtual monoenergetic imaging algorithm, likewise shifting the highest CNR values virtually to the lowest keV levels comparable to our data [12, 20].

Contrary to other DECT approaches, SDCT facilitates the simultaneous measurement of spatially and temporally perfectly aligned high and low energy projection datasets in the upper and lower detector layer and thus can utilize the noise anti-correlation between the detector layers for noise suppression [19]. Moreover, SDCT allows the implementation of recent iterative reconstruction techniques, as high and low energy projection datasets are used for conventional spectral reconstruction leading to a further reduction in image noise [21, 22].

Previously published data reported highest objective image quality parameters and scores of subjective image quality simultaneous far away of the k edge of iodine at 70–80 keV [5, 8]. However, recent technical innovations achieved a separation of both parameters finding the highest CNR and SNR values at 40 keV reconstructions, whereas subjective image quality was rated best in 70 keV reconstructions [12, 20]. In our study, CNR and SNR climax and the best subjective rating of image quality are unified again but at the desired low keV levels. Analogue to SNR/CNR values, the highest scores of subjective image noise and image quality were found in the 40 keV MonoE reconstructions, if window settings could be adjusted according to the reviewers´ preferences.

Without adjustment of the window setting the 70 keV MonoE reconstruction level yielded the lowest subjective image noise and highest subjective image quality in arterial and venous phase imaging. This phenomenon is mainly caused by a disproportionally high attenuation of iodine-containing structures at low keV MonoE reconstructions in standard abdominal windowing presets. In this aspect, we recommend 40 keV MonoE reconstruction levels for interpreting abdominal imaging in arterial and venous phase with individual adjustment of the window settings in addition to conventional images.

One limitation of this study is that only image quality was assessed objectively and subjectively rather than investigating pathologies. However, this study´s aim was to be a technical investigation of a novel scanner technology and, to the authors`knowledge, is the first that provides quantitative and qualitative data derived by SDCT in patients.

In addition, it is worth to mention that quantitative noise measurements are not exactly technically correct in the setting of nonlinear iterative reconstruction techniques, although no better alternative is available.

In conclusion, low keV MonoE (40 and 50 keV) derived from a novel SDCT showed significantly increased CNR as well as SNR values in abdominal vascular and parenchymal structures due to both, increased attenuation values and consistently low image noise values even at the lowest keV levels. Therefore, the key findings of this study using the new detector-based dual energy concept are the low image noise values over the whole energy spectrum from 40 to 200 keV improving the quantitative and qualitative image quality especially at low keV reconstructions.

## Supporting information

S1 File Complete data. (XLSX)
